# Infection prevention and control measures and tools for the prevention of entry of carbapenem-resistant *Enterobacteriaceae* into healthcare settings: guidance from the European Centre for Disease Prevention and Control

**DOI:** 10.1186/s13756-017-0259-z

**Published:** 2017-11-15

**Authors:** A. P. Magiorakos, K. Burns, J. Rodríguez Baño, M. Borg, G. Daikos, U. Dumpis, J. C. Lucet, M. L. Moro, E. Tacconelli, G. Skov Simonsen, E. Szilágyi, A. Voss, J. T. Weber

**Affiliations:** 1 European Centre for Disease Prevention and Control, Stockholm, Sweden; 2 Beaumont Hospital, Royal College of Surgeons in Ireland & Health Protection Surveillance Centre, Dublin, Ireland; 3 Unidad Clínica de Enfermedades Infecciosas y Microbiología, Hospital Universitario Virgen Macarena / Universidad de Sevilla / Instituto de Biomedicina de Sevilla (IBiS), Seville, Spain; 4 Departments of Infection Control & Sterile Services, Mater Dei Hospital, MSD2090, Msida, Malta; 5 First Department of Medicine, Laikon General Hospital, Athens, Greece; 6 Department of Infectious diseases and Infection Control. Pauls Stradins University Hospital, Riga, Latvia; 7 Infection Control Unit, Bichat Claude Bernard Hospital, AP-HP, Paris, France; 8 Agenzia Sanitaria e Sociale Regione Emilia-Romagna, Bologna, Italy; 9 Division of Infectious Diseases, Department Internal Medicine 1, DZIF Center, Tübingen University, Tübingen, Germany; 10 Department of Microbiology and Infection Control, University Hospital of North Norway, and UiT – The Arctic University of Norway, Tromsø, Norway; 11 Department of Epidemiology and Hospital Hygiene, National Public Health and Medical Officer Service, Budapest, Hungary; 12 Department of Medical Microbiology, Radboud University Medical Centre and Canisius-Wilhelmina Hospital, Nijmegen, The Netherlands; 13 Division of Healthcare Quality Promotion, National Center for Emerging and Zoonotic Infectious Diseases, Centers for Disease Control and Prevention, Atlanta, GA USA

**Keywords:** Carbapenem-resistant *Enterobacteriaceae*, CRE, Multidrug-resistant *Enterobacteriaceae*, MDR-E, Antimicrobial resistance, AMR, Healthcare-associated infections, Active screening, Core measures, Supplemental measures

## Abstract

**Background:**

Infections with carbapenem-resistant *Enterobacteriaceae* (CRE) are increasingly being reported from patients in healthcare settings. They are associated with high patient morbidity, attributable mortality and hospital costs. Patients who are “at-risk” may be carriers of these multidrug-resistant *Enterobacteriaceae* (MDR-E).

The purpose of this guidance is to raise awareness and identify the “at-risk” patient when admitted to a healthcare setting and to outline effective infection prevention and control measures to halt the entry and spread of CRE.

**Methods:**

The guidance was created by a group of experts who were functioning independently of their organisations, during two meetings hosted by the European Centre for Disease Prevention and Control. A list of epidemiological risk factors placing patients “at-risk” for carriage with CRE was created by the experts. The conclusions of a systematic review on the prevention of spread of CRE, with the addition of expert opinion, were used to construct lists of core and supplemental infection prevention and control measures to be implemented for “at-risk” patients upon admission to healthcare settings.

**Results:**

Individuals with the following profile are “at-risk” for carriage of CRE: a) a history of an overnight stay in a healthcare setting in the last 12 months, b) dialysis-dependent or cancer chemotherapy in the last 12 months, c) known previous carriage of CRE in the last 12 months and d) epidemiological linkage to a known carrier of a CRE.

Core infection prevention and control measures that should be considered for all patients in healthcare settings were compiled. Preliminary supplemental measures to be implemented for “at-risk” patients on admission are: pre-emptive isolation, active screening for CRE*,* and contact precautions. Patients who are confirmed positive for CRE will need additional supplemental measures.

**Conclusions:**

Strengthening the microbiological capacity, surveillance and reporting of new cases of CRE in healthcare settings and countries is necessary to monitor the epidemiological situation so that, if necessary, the implemented CRE prevention strategies can be refined in a timely manner. Creating a large communication network to exchange this information would be helpful to understand the extent of the CRE reservoir and to prevent infections in healthcare settings, by applying the principles outlined here.

This guidance document offers suggestions for best practices, but is in no way prescriptive for all healthcare settings and all countries. Successful implementation will result if there is local commitment and accountability. The options for intervention can be adopted or adapted to local needs, depending on the availability of financial and structural resources.

**Electronic supplementary material:**

The online version of this article (10.1186/s13756-017-0259-z) contains supplementary material, which is available to authorized users.

## Introduction

Infections with carbapenem-resistant *Enterobacteriaceae* (CRE), and confirmed carbapenemase-producing *Enterobacteriaceae* (CPE) are increasingly being reported from patients in healthcare settings and the community [1–3]. While community and healthcare infections can be caused by CRE and CPE, the epidemiology differs amongst bacterial species, each having a tropism for harbouring certain resistance mechanisms [4].

Infections with CRE and CPE are more difficult to treat, because few, and in some cases no, antimicrobials remain effective against them, due to their extensive resistance patterns. Furthermore, they are associated with high patient morbidity, attributable mortality, and hospital costs [5]. Since *Enterobacteriaceae* are ubiquitous in nature and human gastrointestinal tracts, CRE and CPE are by now dispersed throughout the ecosystem, resulting in community and healthcare infections [6].

### Terms, definitions and tables used in this guidance

**Fig. 1:**
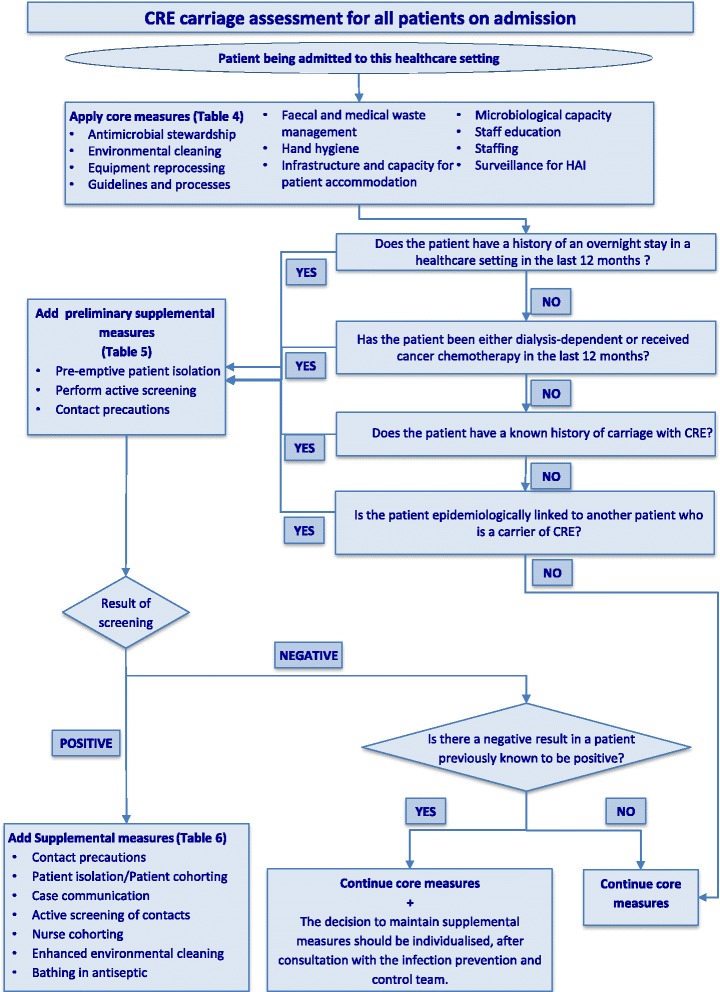
Flowchart for assessment of carriage of carbapenem-resistant *Enterobacteriaceae* in patients being admitted to healthcare settings. **Instructions for use of flowchart in Figure 1 for the management of “at-risk” patients being admitted to healthcare settings** This guidance document was created as a practical tool, for use by frontline HCWs and IPC and control professionals, for the evaluation and management of patients admitted to a healthcare setting. The goal is to identify the “at-risk” patients carrying CRE and to implement measures to prevent the transmission of these bacteria to other patients in the healthcare setting. On admission to the healthcare setting, frontline HCWs should evaluate all patients to see whether they fall into any one of the four risk categories outlined in Table 3 and the flowchart in Fig. 1, and whether they have prior microbiological evidence for CRE carriage. See flowchart on how to manage patients who are potential carriers. All admitted patients should have core measures applied regardless of their carrier status. These should be continued for the duration of their stay. Any patient who is a potential carrier should have the following three preliminary supplemental measures implemented: a) pre-emptive isolation in a single room while waiting for results of screening b) active screening for CRE by obtaining swabs from rectal or perirectal areas and any other site that is either actively infected or considered to be colonised c) contact precautions implemented and used by anyone entering the room. If the result of the active screening is positive for CRE, the measures (patient isolation and contact precautions) are continued and additional supplemental measures are added. Timely communication of the latest microbiological results with the clinical and IPC teams is critical, the patient’s contacts should be screened for CRE carriage, enhanced environmental cleaning applied and consideration given to designated nurse cohorting, based on the clinical situation and location. If the results of active screening are negative for CRE and there is no other indication to continue contact precautions (e.g., patient colonised with another MDRO or patient with a transmissible infection, such as C. difficile) contact precautions can be discontinued, but core measures should be continued. For the patient with a previous positive result for CRE, but from whom CRE is not detected on readmission screening, the decision to continue supplemental measures should be based on a case-by-case risk assessment, in consultation with the IPC team. Factors to be taken into consideration include: the clinical area to which the patient is admitted (e.g., critical care, transplant, oncology), patient age, underlying comorbidity, invasive device use, skin breaks, incontinence, recent antimicrobial use, microbiological tests and schema used for assessing carriage, taking into account the possibility of a false negative screening test result, and interval since the last positive culture for CRE, among others

- For the purposes of harmonisation with other guidance documents and reports in the literature, carbapenem-resistant *Enterobacteriaceae* (CRE) and carbapenemase-producing *Enterobacteriaceae* (CPE), are both referred to as CRE in this document. Where necessary to specify that carbapenem resistance is due to a carbapenemase, however, the term CPE is used.
- The measures suggested in this guidance document are not limited to CRE, but may also be applicable to any species of multidrug-resistant *Enterobacteriaceae* (MDR-E). The decision whether to apply to other MDR-E will be left to local decision-makers and experts.
- The terms “rectal” and “faecal” “carriage” are used in the literature interchangeably with the term “colonisation”. In this guidance document, the term “carriage” is used as an umbrella term to include both “colonisation” and “clinical infection”.
- The term “healthcare facilities” refers to any type of acute and long-term care facilities.
- The term “long-term care facility” (LTCF) refers to any of the heterogeneous types of facilities which “provide delivery of a broad range of services and assistance to people who are limited in their ability to function independently on a daily basis, i.e. to autonomously perform the basic activities of daily living, over an extended period of time. Long-term care comprises a mix of both health and social components, therefore pertaining to both health and social sectors” [7]. These can include “nursing homes, skilled nursing facilities, assisted living, rehabilitation facilities, residential homes, long-term psychiatric facilities” [8]. Countries can define their facilities as LTCFs, by applying and interpreting the general definition provided here.
- Contact precautions include: patient placement, gowns/aprons, gloves, patient transport, disposable noncritical patient-care equipment/patient-dedicated use of such equipment and environmental measures [9].
- In this guidance document, the maximum duration of carriage for “at-risk” patients post discharge is considered to be 12 months. In situations when more than 12 months have elapsed, the admitting healthcare worker (HCW), in consultation with the infection prevention and control (IPC) team, shall decide whether to consider a patient “at-risk”.
- Active screening of “at-risk” patients on admission to a healthcare setting, encompasses rectal screening, as well as screening from any other site which is either actively infected, e.g. draining wounds, or considered to be colonised.
- This guidance document includes six tables (Tables 1, 2, 3, 4, 5 and 6) and a flowchart (Fig. 1). Tables 3, 4, 5, and 6 contain answers to the three main questions posed in this guidance document. The flowchart is a tool to assist frontline workers and IPC teams in their evaluation and decision-making when admitting patients to healthcare settings.
- This guidance document includes printable tables, which can be found in (Additional file 1). These include Table 1, and summaries of Tables 3, 4, 5, and 6 from the main text, as well as a printable version of the flowchart with instructions for use.

### Carbapenem-resistant *Enterobacteriaceae*

Carbapenemases are β-lactamases that hydrolyse carbapenems, usually along with other β-lactams [10]. The most frequently occurring species of *Enterobacteria*ceae which are found to be carbapenem-resistant and that produce carbapenemases, are *Klebsiella pneumoniae* (*K. pneumoniae*) and *Escherichia coli* (*E. coli*) [2]. Carbapenem-resistant *Enterobacteriaceae* display various resistance profiles, depending on the type of genetic elements they harbour and the type of carbapenemases they produce [11]. Since the first report of a carbapenemase in a *K. pneumoniae* isolate harbouring a *Klebsiella pneumoniae* carbapenemase (KPC) in 1996, CPE have now spread globally [12].

**Table 1:** Examples of the most frequently encountered carbapenemases [67]

Acronym	Name or type	First isolated
KPC	*Klebsiella pneumoniae* carbapenemase	1996
VIM	Verona integron-encoded metallo-β-lactamase	1997
OXA-48	OXA-type carbapenemase	2001
NDM	New Delhi metallo-β-lactamase	2008

In Europe, data on carbapenem resistance in *Enterobacteriaceae* are available from various large surveillance networks and projects. Carbapenem resistance in invasive *K. pneumoniae* isolates is collected annually by EARS-Net, and is reported as such, without further description of whether carbapenemases are present [3]. While carbapenem resistance in the EU/EEA remained low in 2015 [3], the population-weighted mean demonstrated a significantly increasing trend from 6.0% in 2012, to 8.1% in 2015 [3]. Similar increases have been shown in other reports [13].

In 2013, the European Centre for Disease Prevention and Control (ECDC) launched the “European survey of carbapenemase-producing *Enterobacteriaceae* (EuSCAPE)”, using a self-assessment tool. The purpose was to describe the European epidemiology of CPE, as well as to increase awareness of and build laboratory capacity for the diagnosis and surveillance of CPE in Europe [14]. The survey was repeated in 2015, with the findings demonstrating that CPE is more widespread than previously thought and is expanding [1]. In 2015, 13 of 38 countries reported either CPE inter-regional spread or endemicity, which was an increase from six countries in 2013. While the repeat survey findings in 2015 demonstrated an increased awareness of the spread of CPE, along with improved microbiological capacity for diagnosis, only 25 (66%) of 38 participating countries had created a functional warning system, with mandatory notification of CPE cases to health authorities [1].

Recent data from the EuSCAPE project provided the first comparable and quality-controlled data on the occurrence of the most important carbapenemases in carbapenem non-susceptible *K. pneumoniae* and *E. coli* clinical isolates from sentinel hospitals in 34 European countries, Turkey and Israel [2]. The results showed the presence of all types of carbapenemases in the collected specimens and a wide variation in geographical distribution of all types of carbapenemases across the countries. Carbapenemases were present in 71% and 40% of the *K. pneumoniae* and *E. coli* non-susceptible isolates, respectively, and the ratio of carbapenemases present in *K. pneumoniae* and *E. coli* was found to be 11 to 1, demonstrating the probable predilection of carbapenemases for *K. pneumoniae* [2].

In this guidance document, for the purposes of harmonisation with other guidance documents and reports in the literature, CRE and CPE will both be referred to as CRE, despite the fact that some CPE may not meet the criteria for resistance to some of the carbapenems, according to present breakpoints from The European Committee on Antimicrobial Susceptibility Testing (EUCAST) [15]. Where necessary to specify, however, CPE will be used.

### Carriage of CRE

Patients who are carriers of, or have clinical infections with CRE can act as reservoirs for transmission to other patients, resulting in carriage, infection or outbreaks. The terms “rectal” and “faecal” “carriage” are used in the literature, interchangeably with “colonisation”. In this document “carriage” will be used as an umbrella term to include “colonisation” and “clinical infection”, since carriers are prone to develop clinical infection [16].

**Table 2:** Examples of select risk factors for carriage of CRE

Patient transfer between healthcare settings within the same country	[45, 68]
Patient transfer between healthcare settings across borders	[20, 21]
Prior admission to an acute care facility	[20, 43, 69]
Prior admission to a LTCF	[34, 45, 69, 70]
Household transmission from patients discharged from healthcare settings	[47]
Foreign travel (e.g. recreational and medical tourism)	[19, 21, 48, 71]

Reliable data regarding the prevalence of CRE carriage from the community and risk populations are not available, and those that are reported most likely represent an underestimation of the true prevalence. This is due to many factors, including but not limited to, differences in the populations and healthcare settings in the studies, as well as the methodology used for screening and reporting. More specifically most of these data come mainly from non-systematic reporting of faecal carriage from active patient screening in various epidemiological settings, e.g. on admission, during outbreaks, during stays at healthcare settings, after discharge from an acute care facility or a LTCF, screening healthy people in the community and pre- and post- foreign travel (Table 2). This renders the data difficult to compare and use in order to create a reliable portrait of CRE carriage.

Until now, data from surveillance networks such as EARS-Net in Europe, along with surveillance data from other areas of the world, plus country reports of outbreaks, permitted us to categorise countries as either “low” or “high” incidence. The resistance landscape has now changed and it has become difficult to be sure of the exact epidemiology of hospitals and countries since it is ever-changing and surveillance studies likely under-detect and report true numbers. This is evident from surveys like EuSCAPE [1, 14] which revealed that countries that were thought to be “low-incidence” may have a higher burden than what was assumed [1]. Even a “low-incidence” country can report CRE, either from imported cases, or even autochthonous cases, leading to hospital outbreaks and even inter-hospital and regional spread [17, 18].

Travel to areas with high prevalence of CRE is also a risk factor for carriage, as is shown in before-and-after travel studies [19]. Undetected carriers present a real challenge and an undetected reservoir for transmission of CRE, because their carrier status is not known and IPC measures are not implemented.

Globally, there has been an increase in the movement of peoples and populations across country borders for reasons which include, tourism (also medical tourism), migration, medical repatriation, and the receipt of healthcare. Patients who are transferred between healthcare settings across borders, or admitted to a healthcare setting while they are abroad, may have become carriers of CRE and can be considered a reservoir of these bacteria, introducing them into other healthcare systems [20, 21]. This risk becomes relevant for any movement of patients, and anyone who enters a healthcare system from across borders anywhere in the world. In the EU, the European Parliament and Council have published Directive 2011/24/EU on the application of patients’ rights in cross-border healthcare [22], essentially facilitating the movement of peoples for the receipt of healthcare. Increased awareness of the risk of carriage of CRE across borders is needed.

## Objective

The purpose of this guidance is to raise awareness of the need to identify the “at-risk” patients who may be a carrier of CRE when they are admitted to a healthcare setting. Furthermore, it will provide practical tools for frontline healthcare workers to evaluate for and screen “at-risk” patients and implement effective IPC measures, when necessary. This document offers suggestions for best practices, but is in no way prescriptive for all healthcare settings or countries. It can be adopted or adapted to local needs, depending on the availability of financial and structural resources.

## Methods

### A systematic review

A systematic literature review was rigorously performed and published by ECDC, to identify the best available evidence on the effective infection prevention and control measures to prevent the transmission of CPE into healthcare settings [23]. The initial population included in the systematic literature search was set to patients who were transferred across borders. This population was subsequently broadened to also include patients admitted to, or transferred between any type of healthcare setting.

To inform this guidance the 2011 ECDC systematic review on the prevention of CPE was updated [20]. Search strategies were not restricted by study design, language or publication status and the analyses were limited to those studies which reported sufficient information to meet items 9 and 17 (intervention description and outcome assessment, respectively) of the ORION statement [24]. The literature search for the systematic review was finalised in July 2013. The Downs and Black criteria [25] were used for quality assessment of observational studies. For comparative (i.e. had two study arms each with a different intervention), the Cochrane Collaboration criteria were used [26]. The effective infection control measures derived from the included studies are listed as conclusions of the systematic review (please see Appendix A) [23].

### Expert meetings

After performing the systematic review, ECDC hosted two meetings of external experts who were functioning independently of their organisations, who evaluated the methodology of the review, provided input on missing studies, and agreed on the level of evidence, suggestions, and guidance structure.

### Questions addressed in the guidance

The expert group agreed to structure the guidance in order to address three main questions, listed below:

1. Which patient groups are considered “at-risk” for carriage of CRE upon admission to a healthcare setting? (Table 3)
2. What are the core IPC measures that should be implemented for all patients in a healthcare setting, regardless of their carrier status for CRE? (Table 4)
3. What are the supplemental, targeted measures that should be implemented, when “at-risk” patients are admitted to healthcare settings, in order to prevent entry and spread of CRE? (Tables 5 and 6)

### Creating the tables and a flowchart

At the expert meeting, the conclusions of the systematic review for CPE were included in this guidance as effective measures for the prevention of entry and spread of CRE into healthcare settings.

From the list of effective measures, the expert group selected relevant measures and populated three other lists containing “bundles” of measures; one bundle for “core measures” (Table 4) and two for “supplemental measures” (Tables 5 and 6). Additional measures considered effective by the expert group were added to these lists as expert opinion. Separating the measures into core and supplemental “bundles” of measures was possible because in the primary studies each measure had been implemented only as part of a bundle and the magnitude of effect of individual measures could not be measured. Selecting the appropriate measures from the original list and placing them in lists containing core and supplemental measures was performed based on measures that were already accepted internationally as “standard precautions” [9], “contact precautions” [9], and “additional precautions” [27], respectively. The source of each measure included in Tables 4, 5 and 6 is designated either as “systematic review” (SR) or “expert opinion” (EO).

This guidance includes six tables (Tables 1, 2, 3, 4, 5 and 6) and a flowchart (Fig. 1). Tables 3, 4, 5, and 6 contain answers to the three main questions posed in this guidance. The flowchart is a tool to assist frontline workers and IPC teams in their evaluation and decision-making when admitting patients to healthcare settings.

## Screening, detection, and management of patients “at-risk” for CRE carriage

Identifying “at-risk” carriers of CRE by evaluating whether they fall into any one of the four risk categories outlined in Table 3, should be performed on admission to the healthcare setting by the frontline healthcare worker admitting the patient. Core measures (see Table 4), which include standard precautions, should be applied to all patients admitted to a healthcare setting, regardless of known or suspected CRE carrier status.

The following preliminary supplemental measures found in Table 5, should be implemented for all patients who are “at-risk” for carriage of CRE:

- Pre-emptively isolated in a single room;
- Active screening for CRE by obtaining swabs from rectal or perirectal areas, and any other site that is either actively infected or considered to be colonised
- Contact precautions implemented and used by anyone entering the room

The following scenarios need to be considered, depending on the results of active screening:

- If the screening test result is positive for CRE, patient isolation and contact precautions are continued, with the addition of supplemental measures in Table 6.
- If the screening test result is negative for CRE, consideration may be given to discontinuing patient isolation and contact precautions, unless there is an indication for their continuation, e.g. colonisation with a different multidrug-resistant organism (MDRO) or transmissible infection. Core measures are maintained for all patients at all times.
- If a patient has a history of CRE carriage, followed by a negative screening test result, the decision on discontinuation of patient isolation and contact precautions remains an open issue. The decision should then be based on a case-by-case risk assessment undertaken by a senior decision maker, in conjunction with advice of the IPC team. Two main concerns contribute to this uncertainty:
  - The possibility of having a false negative result. False negatives can occur due to the lack of standardised protocols for sampling and microbiological testing, as well as issues, such as sampling errors and prior use of antimicrobials, amongst others. A varied approach can be seen in studies that examine duration of carriage and microbiological cultures and PCR are used alone, or in various combinations, as well as with different lengths of time between testing, e.g. two or three negative screening cultures taken a week apart [27–32].
  - Even after documenting clearance of carriage, recurrences have been reported either by failure of initial clearance, or by re-acquisition, making it even more difficult to know whether a patient is no longer a carrier [28, 31].
- The duration of CRE carriage is unknown and multi-factorial. The possibility of false negative test results warrants consideration, as part of the decision to discontinue supplementary precautions.

## Microbiological methods for the detection of CRE

Optimal samples to actively screen for CRE include, specimens of faecal material, as well as active infection sites, e.g. draining wounds, as well as other sites that may be considered colonised, to be defined on a case-by-case basis (e.g., areas of skin breakdown and endotracheal tube aspirates or sputum if the respiratory tract is considered a reservoir). Since faecal specimens are logistically more challenging to obtain, rectal and perirectal swabs are taken instead and have been shown to correlate well with the sensitivity of faecal specimens [33].

To date, there is no consensus on the optimal microbiological methods for detection of carriage of CRE and providing recommendations on microbiological methods is beyond the scope of this document. A sensitive method which provides results in a timely manner is advisable, to facilitate prompt implementation of IPC measures.

## Epidemiological exposures that place patients “at-risk”

Patients may have had epidemiological exposures to situations and environments that can place them “at-risk” for CRE carriage (Table 3). Obtaining a careful medical and travel history is required.

Relevant questions during the medical interview should include the following:

- Has the patient had an overnight stay in a healthcare setting in the last 12 months?
- Has the patient been either dialysis-dependent or received cancer chemotherapy in the last 12 months?
- Does the patient have a known history of previous carriage of CRE in the last 12 months? If the patient has a known history of carriage of CRE in the last 12 months, their CRE carrier status should be documented and communicated when they are transferred from one healthcare setting to another. In addition, patients themselves should be informed of their CRE carrier status and they and their relatives or carers, should be empowered to communicate this information on presentation to healthcare settings.
- Has the patient been previously epidemiologically linked to a known CRE carrier?

**Table 3:** Exposures that place patients “at-risk” patients for carriage of CRE

Any patient who has one of following risk factors is “at-risk” for carriage of CRE:	
a. A history of an overnight stay in a healthcare setting in the last 12 months b. Has been either dialysis-dependent or received cancer chemotherapy in the last 12 months c. Known history of previous carriage of CRE in the last 12 months^a^ d. Has been previously epidemiologically linked to a patient known to be a carrier of CRE^b^	

^a^ Microbiological information is obtained from the patient or is documented in patient’s medical records. If duration from previous microbiological confirmation is longer than 12 months, the decision regarding the risk lies with the admitting physician

^b^ e.g. healthcare or household contacts of patients with known history of carriage of CRE

### A history of admission to a healthcare setting in the last 12 months

Healthcare facilities in areas with high incidence or outbreaks of CRE are reservoirs of CRE [34, 35] (Tables 2 and 3, and flowchart in Fig. 1). Patients with a history of an overnight stay in a healthcare setting in the last 12 months and patients who are re-admitted, or transferred between healthcare settings, are by definition “at-risk” and should be screened for CRE on admission.

If a patient has had contact with a healthcare setting abroad, defined as “outside the boundaries of any country” in the last 12 months, it may be helpful when evaluating the patient’s risk, to refer to the latest epidemiological data on CRE from surveillance studies for that country. However, careful evaluation and decision-making is required when either few or no data are available, or are not recent since the epidemiological situation in a country or healthcare setting can change quickly [1]. In these cases it may be prudent to categorise the patient as “at-risk” by default, pending active screening results (Fig. 1). Furthermore, the physician’s decision about whether the patient will be treated as an “at-risk” patient, may be based on other factors specific to the healthcare setting such as, availability of adequate staffing, structural or financial resources. Due to limitations in surveillance and the slow spread of CRE, even in countries with very low prevalence, it is still strongly suggested to evaluate the risks of each individual patient on admission; the decision whether to screen is ultimately up to the admitting physician. The admission of even one patient who is a carrier, but is undetected, can result in spread of CRE.

### Patients who have been either dialysis-dependent or received cancer chemotherapy in the last 12 months

Certain procedures performed in the ambulatory setting can place a patient “at-risk” for carriage of CRE because of the use of contaminated devices, the presence of long-term intravenous catheters, overuse of antimicrobials, immunosuppression and breaches in IPC during the procedure and other activities surrounding them. Two categories of patients should be considered as “at-risk”: those who have been haemodialysis- or peritoneal dialysis-dependent in the last 12 months [36–39] and cancer (haematology and solid-tumour) patients with long-term intravenous catheters who have received cancer chemotherapy in an outpatient setting in the last 12 months (see Table 3) [40].

Another invasive procedure in the ambulatory setting that one may need to consider on a case-to-case basis, is endoscopic retrograde pancreatography (ERCP), which has been implicated in transmitting CRE by contaminated devices that were not correctly re-processed [41, 42]. For the latter, and any other type of invasive procedure in the ambulatory setting, the level of risk for carriage of CRE attributed to patients who report an invasive procedure in an ambulatory setting in last 12 months will depend on factors that have to do with knowledge of local factors. These include the CRE epidemiology and the level of implementation of IPC measures and healthcare infrastructure in the place where the invasive procedure was performed.

### Previous carriage of, or infection with a CRE in the last 12 months

Known CRE carriers should be carefully evaluated. Persistent carriers can be a source of transmission if supplementary measures, including contact precautions, isolation or cohorting, are not applied on readmission. While it is important to determine their current carrier status, the natural history and duration of carriage of CRE is unknown. Of the few available studies of duration of carriage, most have included high-risk populations (e.g. discharges from, or readmissions to, acute care facilities and LTCFs, and returning travellers) [28, 29, 35, 43, 44]. The duration of carriage varied in all studies underscoring the many factors that influence it and the many types and combinations of microbiological and molecular methods used to determine carriage and clearance. In one study of CRE clearance in patients post-acute care discharge, the median time to culture negativity was 387 days and 39% of patients were still carriers at 12 months [29].

The situation is similar for residents of LTCFs, a population that is at increased risk for persistent carriage for many reasons which include, frequent admissions and re-admissions to acute care facilities, residents’ many co-morbidities, a high prevalence of antimicrobial use and frequent dependency on nursing care in LTCFs. Furthermore, adherence of LTCF staff to isolation and other IPC measures may be more difficult, because implementation of these measures can disrupt the daily activities of the residents, affecting their psychosocial functioning. Therefore, LTCF residents can be a reservoir of CRE within all types of healthcare settings [44, 45].

In one study [44], only 17% of residents who were carriers of *Klebsiella pneumoniae* carbapenemase (KPC)-producing *Enterobacteriaceae* had resolution of carriage during a median four-week stay in a LTCFs and half of those were still carriers when readmitted 9 months later. The duration of CRE carriage is influenced by factors such as immunosuppression, presence of indwelling devices, prior stay in healthcare settings, transfer from or readmission to healthcare settings, poor functional status, high co-morbidity index and exposure to antimicrobials [30, 43].

There are no standardised protocols for sampling and microbiological testing to detect resolution of carriage. Most studies use microbiological cultures or polymerase chain reaction (PCR) alone or a combination of the two, in various combinations and time intervals between testing [28–31]. Furthermore, even after documenting clearance of carriage, recurrences have been reported [28, 31], either by failure of clearance, or by re-acquisition, making it even more important to evaluate each patient on a case-by-case basis. A length of follow up or a duration of carriage of up to 12 months is common and is frequently used as a cut-off due to lack of standardisation and consensus [29, 31].

In this guidance, a maximum duration of carriage for “at-risk” patients post discharge will be 12 months. In situations when the duration is longer than 12 months, the admitting HCW, in consultation with the IPC team, shall decide whether to consider a patient “at-risk”.

### Epidemiologically linked to a patient known to be a carrier of CRE

A known CRE carrier, with whom the patient has been in contact either in a healthcare setting or in the household, should be regarded as a close contact. Although there are limited data to define the time of exposure needed to constitute a “close contact”, one Israeli case-control study [46] reported an exposure to a newly diagnosed carbapenemase- producing CRE patient of ≥3 days, as a risk factor for CRE carriage in a hospital setting.

Close household contacts of CRE carriers [47, 48] can act as reservoirs for transmission. This risk factor should be taken into consideration when household contacts of these patients are admitted to healthcare settings. However, the decision to actively screen household contacts of known CRE carriers will depend on case-by-case risk assessment and local protocols.

## Areas of uncertainty for active screening of specific populations

### Should healthcare workers be screened for CRE upon admission to a healthcare setting?

Active screening of HCWs, when admitted as patients, based on their occupation is not currently recommended. Published prevalence estimates of HCW rectal carriage of CRE rectal carriage vary, depending on the country as well as healthcare and epidemiologic setting [49, 50]. It is most probable, however, that even if a HCW were found to be rectally colonised with CRE, the correct application of standard precautions, and in particular hand hygiene, would most likely disrupt the pathway of transmission of CRE from their hands to patients. When a HCW is admitted as a patient to a healthcare setting, they should, however, be assessed in the same way as any other patient (see Table 3). Further studies are necessary to fully understand the potential reservoir and risk as well as transmission dynamics.

### Should all returning travellers from abroad be screened for CRE upon admission to a healthcare setting?

Foreign travel has been shown to be a risk factor for the carriage of CRE even in previously healthy people [19, 48]. If a patient has a history of foreign travel, the decision of whether or not to screen for CRE carriage, will lie with the admitting physician in consultation with the IPC team. Factors to consider during the evaluation include; epidemiology of the region where patient travelled and patient-specific factors such as, co-morbidities, immunosuppression, diarrhoea during travel and exposure to healthcare settings while abroad.

## Core infection prevention and control measures for all patients

These are considered the basic level of IPC measures [9, 51], and should be applied for all patients for the duration of their stay in a healthcare setting. Three of the measures listed in Table 4 have been further selected and are discussed below. These were selected because of the need to increase awareness for strict compliance (e.g. hand hygiene) and to support the creation of certain structures and indicators in healthcare settings (e.g. antimicrobial stewardship and microbiological capacity).

### Antimicrobial stewardship

Antimicrobials are very commonly prescribed for treatment in human medicine, but may be used unnecessarily in up to 50% of cases [52]. It is important that the effectiveness of existing antimicrobials is preserved for the treatment of infections with CRE. Furthermore, the antimicrobial pipeline is running dry, with a deficit in novel antimicrobial development to address the rise in CRE [53].

A definition of antimicrobial stewardship is provided in a recent article from the Transatlantic Taskforce on Antimicrobial Resistance (TATFAR) Expert Panel on Stewardship Structure and Process Indicators [54]. Antimicrobial stewardship programs are “coordinated programs that implement activities to ensure appropriate antimicrobial prescribing”. In any healthcare setting, antimicrobial stewardship should be implemented as part of a multimodal and integrated approach, along with the application of IPC measures and the invaluable support of a microbiology laboratory that has the capacity for timely and accurate detection of CRE [55].

Antimicrobial stewardship programmes should be multidisciplinary, with a core team made up of an infectious disease physician or clinical microbiologist, and a clinical pharmacist with training in infectious diseases [54]. In order to develop and implement a local antimicrobial stewardship program, it is important that the advice of specialists with expertise in diagnosis, management and prevention of infection is available to all types of healthcare settings including acute, primary and residential care.

### Hand hygiene

Hand hygiene is a core element of standard precautions and the cornerstone to prevent transmission of CRE. To effectively promote hand hygiene, interventions need to be multimodal and sustained over time. Indications and proper technique for hand hygiene and the selection of hand hygiene agents, should be in compliance with the World Health Organization (WHO) recommendations from the WHO guidelines [56].

The hands of HCWs may become contaminated during direct patient care or after contact with the patient’s environment. Hand hygiene has been shown to be the single most effective measure to limit the cross-transmission of MDROs and prevent HAIs [57]. A systematic review conducted by the WHO showed that studies reporting a significant improvement in hand hygiene compliance and/or alcohol based hand rub consumption, also reported a substantial decrease in rates of infections with and/or carriage of MDROs [58]. Although most included studies focused on the transmission of methicillin-resistant *Staphylococcus aureus* (MRSA) as an outcome, the conclusions may apply to CRE.

### Microbiological capacity

Healthcare settings should have access to microbiology laboratories with competence and capacity to detect CRE in both clinical and screening samples. Results from microbiological analyses should be communicated to the clinical staff in a timely manner, and clinically or epidemiologically significant findings should prompt immediate and direct contact with the clinical staff and IPC team to ensure proper follow-up. Microbiological analyses should be performed in accordance with internationally recognized standards, i.e. EUCAST [59] or The Clinical and Laboratory Standards Institute (CLSI) [60], including procedures for quality control. Specific algorithms for microbiological screening and processing of clinical samples may need to be adapted to local epidemiology of antimicrobial resistance, therapeutic traditions, and IPC protocols. All microbiological laboratories should be linked to reference laboratories and participate in external quality assurance schemes for antimicrobial resistance.

**Table 4:** Core infection prevention and control measures to minimize risk of spread of CRE within and between healthcare settings

Intervention (Evidence source)	Comments on measure and implementation
Antimicrobial stewardship (SR)	✓ Healthcare settings should have a formally defined antimicrobial stewardship programme for assuring appropriate antimicrobial use [54] ✓ Healthcare settings should have facility-specific treatment (and prophylaxis) recommendations, based on national guidelines and local microbial susceptibility, to assist with empiric antimicrobial selection [54] ✓ Should be part of a multimodal, integrated programme, along with IPC
Environmental cleaning (SR)	✓ Responsibilities for environmental cleaning and equipment reprocessing must be well-defined and described in hospital internal procedures ✓ Hospitals should review the processes for environmental cleaning and equipment reprocessing, follow instructions of manufacturers, and consider screening (or auditing) to ensure quality of processes
Equipment reprocessing (SR)	
Faecal and medical waste management (EO)	✓ Adequate toilet facilities should be available for all patients ✓ When patients are incontinent or have diarrhoea, bedpans or commodes may be indicated
Guidelines and processes (EO)	✓ Adherence to evidence-based guidelines, processes and pathways for the prevention of healthcare-associated infections (EO)
Hand hygiene (SR)	✓ There is evidence for the effectiveness of hand hygiene, as part of a multimodal strategy, for the reduction of transmission of MDROs [56–58] ✓ Patients should be encouraged to perform hand hygiene, as suggested by WHO guidelines [58]
Infrastructure and capacity for patient accommodation (EO)	✓ Healthcare managers should ensure that the ward occupancy does not exceed the capacity for which it is designed [72] ✓ Healthcare managers should ensure that infection prevention and control building recommendations are followed
Microbiological capacity (EO)	✓ Healthcare settings should have access to microbiology laboratories with capacity to detect CRE from both clinical and screening specimens ✓ Healthcare settings should have systems in place to ensure that potentially significant results are communicated by the microbiology laboratory in a timely manner to the relevant staff in the healthcare setting ✓ Should be part of a multimodal, integrated programme, along with IPC and antimicrobial stewardship
Staff education (SR)	✓ On-going education and training should be provided to all staff with patient contact, with specific reference to CRE
Staffing (EO)	✓ Staffing, appropriate skill level and workload of frontline healthcare workers must be adapted to acuity of care and the number of pool/agency nurses and physicians minimised [72]
Surveillance (EO)	✓ Routine surveillance of healthcare-associated infections

*SR* Systematic review, *EO* Expert opinion

(Please see Additional file 1: Supplementary Table S2 in the supplementary section, for a printable summary of these measures)

## Supplemental measures for “at-risk” patients

In addition to the core measures listed in Table 4, patients who fall into any of the risk categories in Table 1 should have supplemental measures also applied. Initially, when patients are “possible carriers” the three preliminary supplemental measures listed in Table 5, should be applied. These are: a) pre-emptive isolation in a single room b) active screening for CRE by obtaining swabs from the rectal or perirectal areas, and any other site that is either actively infected or considered to be colonised and c) implementation of contact precautions for use by all persons entering the room [27, 32, 61, 62].

### Pre-emptive patient isolation and decisions on patient placement

The evidence from this and other systematic reviews, suggests that patient isolation in a single room should be the goal to limit CRE transmission. Whenever possible, CRE carriers should be placed in a single room with an *en suite* bathroom, in order to reduce the risk of cross transmission [27, 32, 61, 62]. Infrastructural limitations make this not always feasible, however. A limited number of single rooms and even fewer *en suite* isolation rooms, as well as limited financial and staffing resources, e.g. fewer medical staff and IPC professionals, are particularly challenging issues. Resources may be stretched even more when facilities have high rates of CRE*.* The first point prevalence survey of HAI and antimicrobial use in acute care hospitals in the EU/EEA, took place in 2011–2012, reporting a median proportion of single room accommodation in Europe of 9.9% and was less than 5% for eight countries [63].

Whenever a single room is not available, the patient placement decision should be made after an individualised risk assessment performed in consultation with the IPC team. When single rooms are unavailable, cohorting patients who are positive for the same MDROs in the same multi-bedded room is recommended. Prioritisation of bacteria of particular concern, e.g. extensively-drug resistant (XDR) bacteria [64] should also be considered and cohorting of affected patients initiated. Additional attention to cleaning measures and waste management is needed [65]. Whenever possible, nurse cohorting should also be implemented, i.e., designation of nursing staff to care only for the MDRO positive patients during the same shift.

When the option of single room isolation and patient cohorting is available, the decision on placement should be based on an individualised risk assessment. Factors to take into consideration include: the resistance profile of the specific CRE (e.g. the more resistant phenotype takes precedence for single room isolation), known co-colonisation with a different MDRO (e.g., MRSA, VRE) or a transmissible infection (e.g., *C. difficile*) takes precedence for single room isolation and patient factors that pose a higher risk of transmission (e.g., draining wounds, uncontrolled secretions, diarrhoea, incontinence, indwelling medical devices or behavioural issues) [9].

When neither patient isolation in a single room nor cohorting with others carrying the same CRE is feasible, the decision regarding which patients should be put in the same room should again be individualised. Patients with a lower cross-transmission risk may be considered for cohorting (e.g. projected short stay, self-caring, absence of indwelling devices, open or draining wounds and diarrhoea and patients who are continent) [9].

### Contact precautions

For all interactions with the CRE positive patient or resident, contact precautions should be used, in addition to standard precautions [27, 32, 61, 62]. Pre-emptive contact precautions should be implemented for those patients with possible or probable carriage of CRE while awaiting confirmation of the latest status (Fig. 1) in consultation with the IPC team. Periodic audits of compliance with these IPC measures have been linked to favourable outcomes [32].

### Nurse cohorting

Nurse cohorting or designating nursing staff to the care of patients who are positive for MDROs only during their shift, was not studied as a single IPC measure, but was found to be effective in controlling the spread of CRE when implemented as part of a bundle of measures [23, 32]. One can, therefore, expand this term, to include cohorting of other staff groups as well, e.g., doctors and other HCWs, and also use various models to accomplish this, e.g. total cohorting of nursing care or partial daily shift cohorting of medical staff. However, it may be logistically challenging to achieve continuing compliance with staff cohorting in settings where staffing levels are suboptimal at baseline, situations where staffing levels may be reduced (e.g., at night and at weekends) or if there is a low prevalence of CRE patients on the ward.

### Communication on patient/resident transfer within or between healthcare settings

When patients have a positive microbiological result for a CRE, either from active screening or clinical culture, the microbiology laboratory should communicate positive results in a timely manner to the relevant staff and the patient’s healthcare record should be updated with the positive result. Where available, electronic alerts on the facility patient administration IT system may also prompt staff of a patient’s known MDRO status and the need for appropriate placement and IPC precautions. Known positive MDRO status should also be documented and communicated in a timely fashion when patients or residents are transferred between units within the same healthcare setting.

Furthermore, when known CRE carriers are transferred between healthcare settings, inter-facility communication is important to facilitate immediate implementation of appropriate IPC measures, to inform patient placement and the interpretation of local active screening results. Healthcare providers should ensure that the receiving facility is informed of a patient’s CRE carrier status, and that transfer documentation also contains information about any relevant positive microbiology results. It is suggested that the patient’s CRE carriage status could be included as a separate diagnosis. Information about recent local clusters or ongoing outbreaks should also be communicated to the receiving facility.

Since the prevalence of relevant determinants in the average human microbiome of different geographical/societal populations is unknown, it should be assumed that inter-facility patient transfer from any country may pose a risk. Active screening of rectal or perirectal swabs taken on patient transfer is an effective CRE detection method. The European Parliament and Council published Directive 2011/24/EU on the application of patients’ rights in cross-border healthcare [22], which will increase the awareness of the risk of transferring MDROs, including CRE when patients seek healthcare abroad. Developing a standardised pan-European form could be created, which would contain positive microbiological data. This document would be transported by the patient, placed in a visible position in the chart, or sent to the receiving healthcare facility. Additional verbal communication prior to the transfer is encouraged.

Development of EU-wide CRE surveillance, with strengthening of mandatory reporting could be used to generate timely information about prevalence, incidence, and outbreaks of CRE*.*

Rapid exchange of information at the EU level could be facilitated via the Epidemic Intelligence Information System (EPIS) or the Early Warning and Response System (EWRS).

### Active surveillance and contact tracing when patients are epidemiologically linked to others who are CRE carriers

There are situations when inpatient active surveillance and contact tracing may be considered to determine whether or not onward transmission has occurred.

- Detection of CRE from an inpatient who was not isolated or on contact precautions (e.g., detection from a clinical specimen or screening specimen taken on transfer from a ward to a high-risk area where active screening is performed)
- In the event that supplemental precautions had been implemented, but there is evidence of suboptimal staff compliance with recommended precautions
- Active surveillance and contact tracing is suggested in the event of a cluster or outbreak of CRE, with identification potential epidemiological links or invasive procedures [42].

In healthcare settings with low CRE prevalence, the number of contacts to be screened for CRE is determined on a case-by-case basis, taking into account proximity to the index case, duration of exposure, and whether there was shared nursing staff. In “at-risk” units, (e.g. intensive care, transplant surgery, haematology oncology wards) the entire unit may need to be screened for CRE [66]. If additional CRE carriers are identified, the circle of screening will need to be expanded, taking into account the contacts of the newly detected carriers.

**Table 5:** Preliminary supplemental infection prevention and control measures for CRE “at-risk” patients with or without known microbiological results

Intervention (Evidence source)	Comments on measure and implementation
Pre-emptive isolation of patients on admission (SR)	✓ Isolation in single rooms either upon admission or when patients are actively screened for carriage of CRE ✓ Decision for patient placement should be made after individualised risk assessment by, and consultation with the IPC team ✓ *En suite* or bathrooms designated for use by known carriers, or commodes are strongly suggested for all patients on contact precautions for CRE
Active screening on admission (SR)^a^	✓ Active screening of all “at-risk” patients on admission to healthcare setting
Contact precautions (SR)	✓ Contact precautions should be used for direct contact with patient or their environment ✓ *En suite* or bathrooms designated for use by known carriers, or commodes are strongly suggested for all patients on contact precautions for CRE

*SR* Systematic review, *EO* Expert opinion

^a^Active screening encompasses rectal screening, as well as screening from any other site which is either actively infected, e.g. draining wounds, or considered to be colonised

(Please see Additional file 1: Supplementary Table S3 in the supplementary section, for a printable summary of these measures)

**Table 6:** Supplemental infection control and prevention measures for patients with CRE preliminarily positive or confirmed microbiological results

Intervention (Evidence source)	Comments on measure and implementation
Contact precautions (SR)	✓ Contact precautions should be continued when patient is suspected positive or confirmed positive ✓ Contact precautions should be used for direct contact with patient or their immediate surroundings and/or surfaces ✓ *En suite* or bathrooms designated for use by known carriers, or commodes are strongly suggested for all patients on contact precautions for CRE
Patient isolation or patient cohorting (SR)	✓ When patients were previously pre-emptively isolated they should remain isolated if results of active screening are suspected positive or confirmed positive ✓ If not already isolated, the patient should be isolated upon receipt of suspected or confirmed positive microbiological result
Case communication (SR) (Communication about microbiological results within healthcare settings)	✓ Communication on patient/resident transfer within a healthcare setting ✓ Positive results should be communicated by the microbiology laboratory in a timely manner to the relevant staff ✓ Healthcare record flagging and use of patient administration IT system flagging if feasible within healthcare setting, regarding any positive microbiological information ✓ Consider including patient’s carriage or infection status for CRE as a separate diagnosis ✓ Positive microbiological data should be communicated in a timely fashion when patients are transferred between units
	Communication on patient/resident transfer between healthcare settings ✓ Transfer documentation must accompany patient/resident, with information about known carriage or infection status ✓ Consider including patient’s carriage for CRE as a separate diagnosis ✓ Positive microbiological data should be communicated in a timely fashion when patients are transferred between healthcare settings within regions, country and across borders ✓ The responsibility to notify the receiving healthcare setting of patient’s/resident’s relevant microbiological data rests with the referral healthcare setting ✓ Ensure communication by a responsible person of local current or recent clusters or outbreaks to the receiving institution when patients/residents are transferred
	Communication on patient transfer between healthcare settings in different countries ✓ Transfer documentation must accompany patient, with information about patient’s carriage or infection status ✓ Consider including patient’s carriage or infection status for CRE as a separate diagnosis ✓ Recognise the importance of implementing the cross-border Directive^a^ in preventing inter-country spread of CRE ✓ Ensure timely communication with receiving healthcare setting for all positive patient microbiological data ✓ Ensure that patient’s rights for personal data protection are secured when sharing patient data between healthcare settings1
Active screening of contacts (SR)	✓ Active screening of patients/residents who are epidemiologically linked to a known CRE carrier
Nurse cohorting (SR)	✓ While acknowledging existing limitations in staffing and other resources, cohorting or designated nursing staff is strongly suggested for the care of patients with CRE
Enhanced environmental cleaning (EO)	✓ Enhanced cleaning should be performed, especially for areas in close proximity to CRE carriers ✓ Terminal disinfection of rooms should be performed upon transfer or discharge of patients
Bathing in antiseptic (SR)	✓ Data mostly available from Gram-positive organisms; can be used as a horizontal approach for other MDROs [73] ✓ Due to lack of strong evidence, can be considered for use in difficult-to- control situations

*SR* Systematic review, *EO* Expert opinion

^a^DIRECTIVE 2011/24/EU OF THE EUROPEAN PARLIAMENT AND OF THE COUNCIL of 9 March 2011 on the application of patients’ rights in cross-border healthcare [22]

(Please see Additional file 1: Supplementary Table S4 in the supplementary section, for a printable summary of these measures)

## Key messages and conclusions

The global increase in infections with CRE is cause for concern, because these highly resistant bacteria are associated with higher patient morbidity, attributable mortality, and few or no antimicrobials remain effective for treatment. Acute care facilities and long-term care facilities can be reservoirs of CRE and patients who spend time in healthcare settings, have had an invasive procedure in ambulatory care, have had close contact with someone who is a CRE carrier, are at risk for becoming carriers. Carriers can then act as reservoirs for spread of these bacteria when admitted to or transferred between healthcare settings.

When admitted to a healthcare setting, core IPC measures should be implemented for all patients and maintained for the duration of their stay. They are the most basic level of IPC precautions and HCWs should strictly adhere to these to prevent spread of CRE.

In addition to the core measures, patients who fall into any of the risk categories should have supplemental IPC measures applied. Initially, when patients meet the exposure criteria, they are “possible carriers” and three preliminary supplemental measures should be applied: a) pre-emptive isolation of the patient in a single room b) active screening for CRE by obtaining swabs from rectal or perirectal areas, and any other site that is either actively infected or considered to be colonised, and c) implementation of contact precautions for use by all persons entering the room.

Countries and healthcare settings should ensure that they have both the microbiological capacity and sensitive and timely methodology to detect carriage of CRE*.*

Whenever possible, CRE carriers should be accommodated in isolation rooms with *en suite* bathroom facilities. In the face of increasing antimicrobial resistance, local and national strategies are required to modernise healthcare infrastructure to meet the challenges of MDROs.

The availability of an adequate number of appropriately trained IPC healthcare professionals for acute and long-term care healthcare settings, along with appropriate training for all HCWs for the correct implementation of IPC measures, including the core and supplementary measures outlined in this document, is of paramount importance.

When patients have a positive microbiological result, from either active screening or a clinical culture, the microbiology laboratory should communicate positive results in a timely manner to the relevant staff, the patient’s record should be visibly flagged with the positive result, and the positive data should be communicated in a timely fashion when patients are transferred between units.

Furthermore, when CRE carriers are transferred between healthcare settings, healthcare providers should communicate the patient’s status to the receiving facility and transfer documentation should contain information about the positive result. The responsibility for this communication lies with the person sending the patient. Information about recent clusters or outbreaks should also be communicated to the receiving facility.

A larger, effective communication network within countries in the European Union is necessary, so that mandatory reporting of new cases of CRE can be shared with all relevant parties. EU-wide surveillance with strengthening of mandatory reporting can be an option to facilitate the monitoring of these resistant bacteria.

This guidance is not prescriptive for all healthcare settings and countries. Successful implementation of this guidance can only happen if there is local commitment and accountability of this guidance adapted to local needs, taking into account available resources. Furthermore, the dissemination of this guidance among EU/EEA countries will help raise awareness among countries, healthcare authorities and healthcare workers, about the need to identify CRE carriers upon hospital admission. The way this guidance will be used and how the conclusions will be extrapolated to apply to other MDROs will be up to the decision and policy of the local healthcare settings.

## Appendix A. Conclusions of ECDC systematic review on effective infection prevention and control measures for CPE [23]

- In agreement with the 2011 ECDC risk assessment, there is no evidence on infection control measures to specifically prevent the transmission of CRE during cross-border transfer. Two studies included in the updated 2013 review did include patients transferred between hospitals in the same region. From these studies, there is evidence that infection control measures were effective in reducing imported CRE.
- The findings from the updated 2013 review agree with and extend the findings from the 2011 ECDC risk assessment, in that the evidence for the effectiveness of infection control measures comes only from observational studies reporting infection control measures in bundles (evidence level ++). This evidence is limited by the lack of data from controlled studies reporting single infection control measures.
- As in the 2011 ECDC risk assessment, evidence from outbreak reports in acute care settings was identified in this 2013 updated review for the effectiveness of the early implementation of active surveillance by rectal screening for CRE carriage on hospital admission, admission to specific wards/units, and for surveillance during outbreaks (evidence level ++).
- As in the 2011 ECDC risk assessment, evidence was identified in this 2013 review for the effectiveness of: a) pre-emptive isolation on admission, b) dedicated nursing or other staff and c) contact precautions (evidence level ++).
- In this review evidence was identified for the effectiveness of the following infection control measures: a) patient cohorting, b) hand hygiene, c) patient isolation, d) nursing (or staff) cohorting (similar to dedicated nursing), e) environmental cleaning, f) staff education, g) case notification/flagging, h) contact tracing and i) antimicrobial restriction (evidence level ++).
- Evidence for the effectiveness of ward or intensive care unit closure remains available from the original 2011 ECDC risk assessment. No new evidence for these was identified in this 2013 updated review.
- Other infection control measures may also be effective, but the evidence supporting their effectiveness is less clear due to a lack of data.
- The best available evidence for the effectiveness of interventions derived from this review and the 2011 ECDC risk assessment comes from data reported from observational studies, which, for the most part include interventions that are part of a bundle of measures, making the effectiveness of each measure less clear. It would be necessary to strive for better-designed and reported studies that provide evidence for the benefit and harm of infection control measures for the prevention and control of CRE.

## Additional file

Additional file 1: Printable tables and material for frontline workers. (DOCX 1280 kb)
