# Supplementary material for: Infection prevention and control measures and tools for the prevention of entry of carbapenem-resistant Enterobacteriaceae into healthcare settings: guidance from the European Centre for Disease Prevention and Control
Source: Antimicrob Resist Infect Control. 2017 Nov 15;6:113. doi: 10.1186/s13756-017-0259-z (PMC5686856; doi:10.1186/s13756-017-0259-z)
Supplement: Additional file 1: — Printable tables and material for frontline workers. (DOCX 1280 kb) [file 13756_2017_259_MOESM1_ESM.docx]

# **Additional file 1**

# **Printable tables and material for frontline workers when patients are admitted to a healthcare setting**

# **Supplementary Table S1.** Exposures that place patients “at-risk” for carriage of CRE

**Any patient who has one of following risk factors is “at-risk” for carriage of CRE:**

1. A history of an overnight stay in a healthcare setting in the last 12 months
2. Has been either dialysis-dependent or received cancer chemotherapy in the last 12 months
3. Known history of previous carriage of CRE in the last 12 months^1^
4. Has been previously epidemiologically linked to a patient known to be a carrier of CRE^2^

^1^ Microbiological information obtained from the patient or is documented in patient’s medical records. If duration is longer than 12 months, the decision regarding the risk lies with the admitting physician.

^2^ e.g. healthcare or household contacts of patients with known history of carriage of CRE.

#

# **Supplementary Table S2.** Core infection prevention and control measures* to minimize risk of spread of CRE within and between healthcare settings

- Antimicrobial stewardship
- Environmental cleaning
- Equipment reprocessing
- Faecal and medical waste management
- Guidelines and processes
- Hand hygiene
- Infrastructure and capacity for patient accommodation
- Microbiological capacity
- Staff education
- Staffing
- Surveillance for HAI

*^*^Should be applied during the entire stay of the patient*

# **Supplementary Table S3.** Preliminary supplemental infection prevention and control measures* for CRE “at-risk” patients with or without known microbiological results

- Pre-emptive isolation of the patient
- Perform active screening^1^
- Contact precautions

*Should be applied in addition to core measures, when patient is “at-risk” and epidemiological status is being investigated

^1^Active screening encompasses rectal screening, as well as screening from any other site which is either actively infected, e.g. draining wounds, or considered to be colonised.

# **Supplementary Table S4.** Supplemental infection prevention and control measures* for “at-risk” patients with CRE preliminarily positive or confirmed positive microbiological results

- Contact precautions
- Patient isolation/Patient cohorting
- Case communication (intra- and intra-hospital and inter-country communication)
- Active screening of contacts
- Nurse cohorting
- Enhanced environmental cleaning
- Bathing in antiseptic

** Should be applied in addition to core measures*

# **Supplementary Figure S1.** Flowchart for assessment of carriage of carbapenem-resistant *Enterobacteriaceae* in patients being admitted to healthcare settings


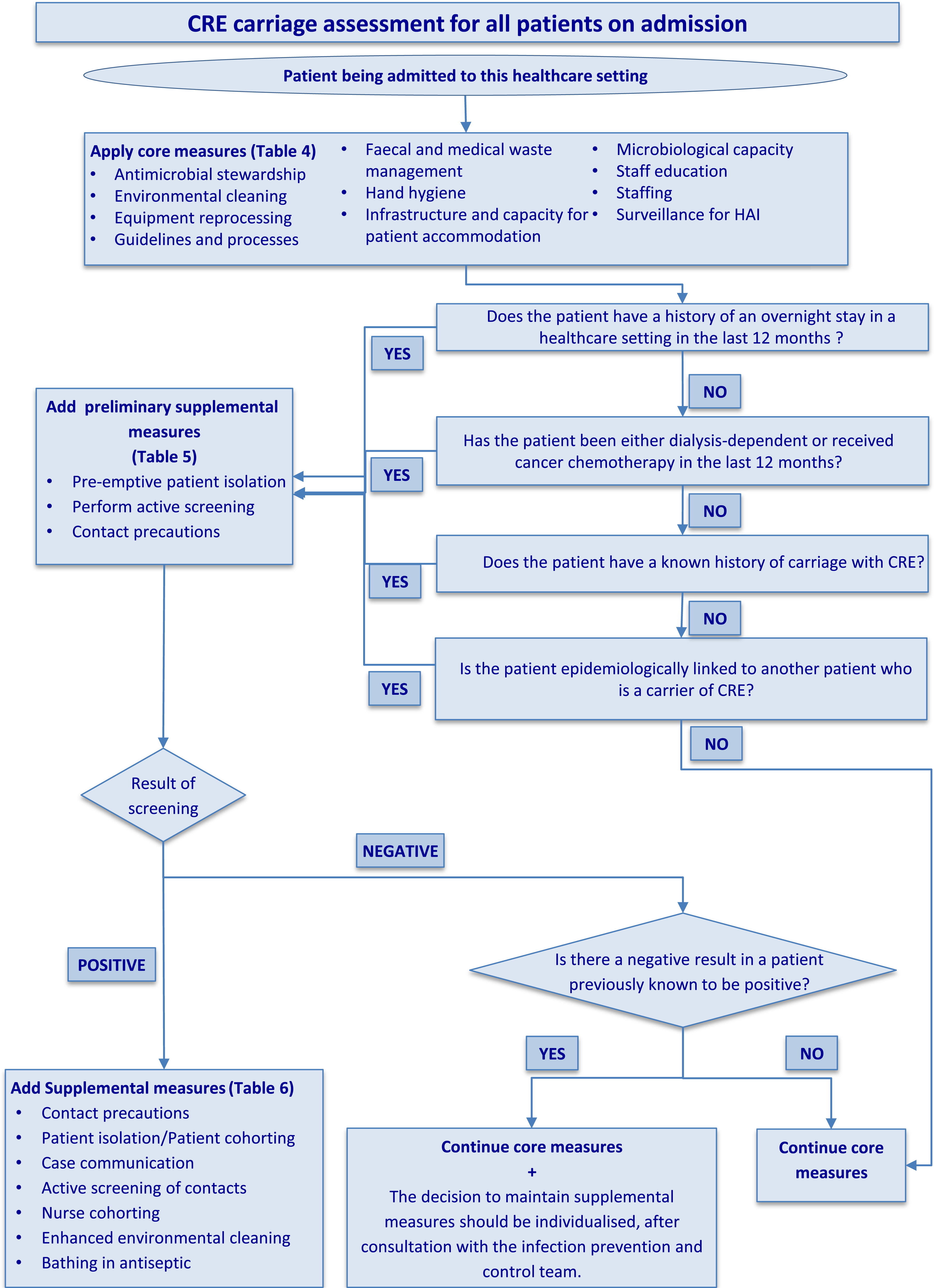


# **Instructions for use of flowchart in Supplementary Figure S1 for the management of “at-risk” patients being admitted to healthcare settings**

This guidance document was created as a practical tool, for use by frontline HCWs and IPC and control professionals, for the evaluation and management of patients admitted to a healthcare setting. The goal is to identify the “at-risk” patients carrying CRE and to implement measures to prevent the transmission of these bacteria to other patients in the healthcare setting

On admission to the healthcare setting, frontline HCWs should evaluate all patients to see whether they fall into any one of the four risk categories outlined in Appendix Table 1 and Appendix Figure 1, and whether they have prior microbiological evidence for CRE carriage. See flowchart on how to manage patients who are potential carriers.

All admitted patients should have core measures applied regardless of their carrier status. These should be continued for the duration of their stay.

Any patient who is a potential carrier should have the following three preliminary supplemental measures implemented:

a) pre-emptive isolation in a single room while waiting for results of screening

b) active screening for CRE by obtaining swabs from rectal or perirectal areas and any other site that is either actively infected or considered to be colonised

c) contact precautions implemented and used by anyone entering the room.

If the result of the active screening is positive for CRE, the measures (patient isolation and contact precautions) are continued and additional supplemental measures are added. Timely communication of the latest microbiological results with the clinical and IPC teams is critical, the patient’s contacts should be screened for CRE carriage, enhanced environmental cleaning applied and consideration given to designated nurse cohorting, based on the clinical situation and location.

If the results of active screening are negative for CRE and there is no other indication to continue contact precautions (e.g., patient colonised with another MDRO or patient with a transmissible infection, such as C. difficile) contact precautions can be discontinued, but core measures should be continued.

For the patient with a previous positive result for CRE, but from whom CRE is not detected on readmission screening, the decision to continue supplemental measures should be based on a case-by-case risk assessment, in consultation with the IPC team. Factors to be taken into consideration include: the clinical area to which the patient is admitted (e.g., critical care, transplant, oncology), patient age, underlying comorbidity, invasive device use, skin breaks, incontinence, recent antimicrobial use, microbiological tests and schema used for assessing carriage, taking into account the possibility of a false negative screening test result, and interval since the last positive culture for CRE, among others.
